# A latent profile analysis of cancer-related fatigue in patients with breast cancer undergoing chemotherapy and its relationship with quality of life: a cross-sectional study

**DOI:** 10.3389/fpubh.2026.1877774

**Published:** 2026-07-07

**Authors:** Lingling Yang, Lina Tong, Liping Zhang, Xi Zhang, Rong Wang, Li Su, Xinyan Wang

**Affiliations:** 1 Department of Surgical Oncology, General Hospital of Ningxia Medical University, Yinchuan, China; 2 School of Nursing, Ningxia Medical University, Yinchuan, China; 3 Department of Nursing, Hospital of Cardio-cerebrovascular Disease, General Hospital of Ningxia Medical University, Yinchuan, China

**Keywords:** breast neoplasms, cancer-related fatigue, chemotherapy, latent profile analysis, quality of life

## Abstract

**Introduction:**

The identification of classes of cancer-related fatigue in patients with breast cancer undergoing chemotherapy is crucial for the implementation of individualized intervention measures. This study aims to determine the demographic and clinical characteristics of each profile through latent profile analysis, and particularly examines the association between different profiles of cancer-related fatigue and the quality of life (QoL) of patients.

**Methods:**

Patients with breast cancer undergoing chemotherapy for Department of Medical Oncology at a tertiary general hospital in Northwest China between April and August 2023 were recruited using convenience sampling. Employed the General Data Questionnaire, the Cancer Fatigue Scale, and the Functional Assessment of Cancer Therapy-Breast Scale to measure cancer-related fatigue and QoL. Latent profile analysis was performed to identify potential classifications of cancer-related fatigue. Univariable and multivariate logistic analysis were used to determine associated influencing factors.

**Results:**

Three latent profiles of cancer-related fatigue were identified: low physical and cognitive fatigue–high emotional fatigue group (24.4%), moderate fatigue group (39.2%), and high physical and cognitive fatigue–low emotional fatigue group (36.4%). Multivariate logistic analysis identified average daily step count during the past week ≤ 4,999 steps (*OR* = 0.214, 95% CI: 0.049~0.940, *P* = 0.041) and KPS score ≤ 70 (*OR* = 6.377, 95% CI: 1.953~20.819, *P* = 0.002) as significant predictors of moderate fatigue group, tumor stage III (*OR* = 0.272, 95% CI: 0.080~0.922, *P* = 0.037) and absence of pain (*OR* = 0.360, 95% CI: 0.180~0.718, *P* = 0.004) as important predictors of high physical and cognitive fatigue–low emotional fatigue group.

**Conclusions:**

There are different characteristic groups of cancer-related fatigue in patients with breast cancer undergoing chemotherapy. Medical staff can formulate personalized intervention plans based on the different types of fatigue characteristics of patients to enhance their QoL.

## 1 Introduction

According to the latest Global Cancer Report 2022, breast cancer as a major public health concern affecting women's health worldwide. It is the second most frequently diagnosed malignancy globally and the leading cause of cancer-related deaths among women (1). The situation regarding breast cancer prevention and control in China is extremely serious. According to the latest data from the National Cancer Center, approximately 357,200 new breast cancer cases are diagnosed annually among Chinese women, representing 15.59% of all new malignant tumor cases in females (2). As the core means of comprehensive treatment for breast cancer, chemotherapy plays a vital clinical significance in consolidating the effect of surgical treatment, preventing recurrence and metastasis, and enhancing the survival rate of patients (3, 4). However, the long-term impact of treatment-related adverse effects on patients' QoL has become increasingly prominent (5), among which cancer-related fatigue has emerged as a critical challenge in clinical care due to its high prevalence and persistent characteristics (6, 7).

Relevant studies have reported that the prevalence of cancer-related fatigue among patients with breast cancer undergoing chemotherapy ranges from 52.07 to 62.10% (8, 9). Furthermore, these fatigue symptoms may persist for months or even years following the completion of chemotherapy treatment (10). Guidelines issued by the National Comprehensive Cancer Network clearly articulate that cancer-related fatigue is closely associated with the malignancy itself and its treatment, and its severity is disproportionate to recent physical activity levels. This condition disrupts patients' normal physiological functions and can trigger multidimensional impairments in their physical, cognitive, and emotional wellbeing (11), and may be detrimental to QOL of patients.

Previous studies regard cancer-related fatigue one-dimensionally by calculating the total or mean fatigue score in the entire sample; they are thus unable to capture distinct within-groups variation in fatigue (12, 13). However, as a subjective individual experience, cancer-related fatigue exhibits significant variability. And individuals with the same total score on the scale may exhibit completely different response patterns in each dimension, reflecting the inherent differences in the structure and intensity of their attitudes (14). Therefore, the typological characteristics of cancer-related fatigue in patients with breast cancer undergoing chemotherapy further study to prescribe more precise and cost-effective interventions.

As an individual-centered statistical approach, latent profile analysis (LPA) can measure the continuous manifest variables and clusters the population into potential subgroups with different characteristic patterns (15). Moreover, unlike traditional methods such as cluster analysis, LPA is based on model fitting estimation, which provides more accurate and objective classification (16). Consequently, the use of LPA enables the identification of significant differences in the characteristics of different profiles of cancer-related fatigue in patients with breast cancer undergoing chemotherapy, thereby facilitating the development of more targeted interventions. In fact, LPA has been employed in several recent studies to capture within-group variation by clustering subgroups of patients with distinct profiles (17–19). Nevertheless, only a few studies have examined different subgroups of patients with distinct fatigue profiles (20–23). For example, Wright et al. (21) identified four distinct morning fatigue profiles (very low, low, high, and very high) among a heterogeneous sample of oncology outpatients receiving chemotherapy. However, this study assessed only physical fatigue, and fatigue profiles were labeled according to symptom severity. Therefore, the application of LPA in the field of cancer-related fatigue research among breast cancer patients undergoing chemotherapy remains relatively limited at present.

As the core evaluation index of health outcomes in cancer patients, QoL is correlated with cancer-related fatigue (24). Existing evidence indicates that cancer-related fatigue can directly or indirectly impair patients' QoL by limiting daily activity ability, exacerbating emotional distress, and reducing social participation (25, 26). Although previous studies have thoroughly explored the predictive role of the overall level of cancer-related fatigue on QoL (24), research examining the impact of different fatigue types on QoL remains relatively scarce. Therefore, more in-depth investigation into the relationship between cancer-related fatigue and QoL in cancer patients is warranted. Rahman et al. (27) assessed the level of cancer-related fatigue in patients with breast cancer and explored its correlation with QoL; however, their study did not conduct a subgroup-specific in-depth analysis of this relationship. Applying LPA to patients with breast cancer undergoing chemotherapy can help reveal the various types of cancer-related fatigue and their correlations with QoL. This, in turn, provides a basis for researchers to develop customized intervention and support strategies based on the characteristics of different fatigue types.

Therefore, guided by existing literature, we propose the following research questions: (a) Are there different latent profiles of cancer-related fatigue among patients with breast cancer undergoing chemotherapy, and what are the differences in the distribution of different profile categories in terms of the demographic and clinical characteristics of the patients? (b) What is the association between different categories of fatigue profiles and patients' QoL and its dimensions? We hypothesize that: (a) Distinct latent profiles of cancer-related fatigue exist, each of which is characterized by unique demographic and clinical characteristics; (b) There is a significant association between different categories of fatigue profiles and QoL and its dimensions. These findings will provide a scientific basis for constructing precise nursing models based on different cancer-related fatigue profile categories, with the aim of improving QoL for patients with breast cancer.

## 2 Methods

### 2.1 Study design

The convenience sampling method was used to select patients with breast cancer who were undergoing chemotherapy in the Department of Medical Oncology of the General Hospital of Ningxia Medical University from April to August 2023 as the participants. The inclusion criteria were as follows: (1) Aged ≥18 years, pathologically diagnosed with breast cancer; (2) Has undergone surgical treatment and has been supplemented with chemotherapy after the operation; (3) Clearly conscious, no verbal communication or writing difficulties. Exclusion criteria: (1) Comorbidities with other primary cancers or severe chronic diseases; (2) Patients with a history of psychiatric disorders and personality disorders. The descriptive study sample size formula $n={\left(\mu_{\alpha /2}\sigma \right)}^{2}/\delta^{2}$ was used, referring to the research results of Meng et al. (28), σ = 7.87, δ = 1.1, α = 0.05, and considering the 20% invalid questionnaire rate, the sample size was at least 236 cases.

### 2.2 Measurement

#### 2.2.1 General data questionnaire

The general data questionnaire, which included the demographic data (age, education level, occupation, working condition, marital status, family place of residence, etc) and clinical characteristics (number of courses of chemotherapy, tumor stage, pathological diagnosis, average daily step count during the past week, etc). The average daily step count during the past week was measured through self-reporting by the patients. The patients were asked to recall and estimate the average number of steps per day over the past seven days. The researchers used standardized instructions for questioning to reduce recall bias.

#### 2.2.2 Cancer fatigue scale

The scale was developed by Okuyama et al. (29) and later translated and locally revised by Zhang et al. (30) to assess the fatigue status of patients undergoing chemotherapy. This measurement tool covers three dimensions: physical fatigue (seven items), emotional fatigue (four items), and cognitive fatigue (four items). Each item is scored on a 0–4 scale, increasing from “no fatigue” to “severe fatigue” in sequence. The total score of the scale ranges from 0 to 60, and the score is positively correlated with the severity of fatigue symptoms. In this study, the Cronbach's α coefficients of the three dimensions of this scale ranged from 0.713 to 0.869.

#### 2.2.3 Functional assessment of cancer therapy-breast scale

The scale used in this study to assess the QoL of patients with breast cancer was translated and adapted into Chinese by Wan et al. (31). The measurement tool consists of five dimensions: physical wellbeing, social and family wellbeing, emotional wellbeing, functional wellbeing, and breast cancer-specific concerns. Each item in the scale is scored on a scale of 0 to 4, ranging from “not at all” to “extremely,” with higher scores indicating better QoL the patients. In this study, the Cronbach's α coefficients for the use of this scale by patients was 0.875.

### 2.3 Data collection

After obtaining consent from the patients at the hospital, researcher conducted face-to-face interviews to collect the data. The purpose and significance of the study were explained to patients before the survey, and informed consent was obtained. Unified guide were used to inform patients about the method of questionnaire completion, either by the patients themselves or by the investigator recording their verbal responses to ensure that no instructive expressions influenced their choices.

After completing the questionnaire, it should be retrieved promptly. Researchers will check the completeness of the questionnaire on the spot. If any missing items or incorrect entries are found, they should promptly ask the patient and make supplementary or correction on the spot. A total of 264 questionnaires were collected from patients wiit breast cancer in this study. After screening, six invalid questionnaires (those with perfunctory responses or incomplete information) were excluded. Finally, 258 valid questionnaires were obtained, with a valid recovery rate of 97.73%.

### 2.4 Ethical considerations

This study has been approved by the Medical Ethics Committee of Ningxia Medical University General Hospital (KYLL-2023-0347). All participants signed a written informed consent form before the start of the study.

### 2.5 Data analysis

The questionnaire database was established by EpiData3.1 software, and the data were entered independently by two researchers. After the double check, we used SPSS 26.0 and Mplus 8.3 Software to perform the statistical analysis of the data.

Conduct LPA on the items of cancer-related fatigue using Mplus 8.3 software. The fit indices mainly include (32, 33): (1) Akaike Information Criterion (AIC), Bayesian Information Criterion (BIC), and adjusted BIC (aBIC). The smaller the values of these three statistical indicators, the better the model fit. (2) Entropy is used to evaluate the accuracy of the model, with values ranging from 0 to 1. The closer the value is to 1, the more accurate the model fit. (3) The Lo-Mendell-Rubin test (LMRT) and bootstrap likelihood ratio test (BLRT) values are significant (*P* < 0.05), indicating that the *k*-class model fits better than the *k*-1 class model. (4) Models with a probability of at least 5% for each category are classified more reasonably. SPSS 26.0 software was used to analyze the data. For measurement data with skewed distributions, the median (quartiles) was used for description and the rank sum test was used for comparisons between groups; for count data, they were expressed in the form of case numbers and percentages, and the chi-square test was used for comparisons between groups. Multivariate logistic analysis was conducted to explore demographic and clinical characteristics that influenced the latent profile of cancer-related fatigue. Evaluate the overall model fit using the likelihood ratio test. Finally, Kruskal–Wallis *H* test and *Post Hoc* Multiple Comparisons was performed to further determine the difference in QoL between different latent profiles of cancer-related fatigue.

## 3 Results

### 3.1 Participant characteristics

A total of 258 patients with breast cancer undergoing chemotherapy were included in this study (Table 1). Of these patients, 258 were female. The mean age of patients was 50.27 ± 8.80 years, 64.0% had completed Junior high school and below, 54.3% were unemployed, 61.2% resided in towns, and 31.8% presented with tumor stage IV.

**Table 1:** Demographic and clinical characteristics of the study participants (*n* = 258).

Item	Frequency	Percentage
Age (years)		
18–44	68	26.3
45–59	156	60.5
≥60	34	13.2
Education level		
Junior high school and below	165	64.0
High school/technical secondary school	56	21.7
University and above	37	14.3
Working condition		
Employed	70	27.1
Unemployed	140	54.3
Retired	48	18.6
Marital status		
Married	233	90.3
Other (single/divorced/widowed)	25	9.7
Family place of residence		
Towns	158	61.2
Village	100	38.8
Per capita monthly household income (¥)		
≤ 2,999	93	36.1
3,000–4,999	126	48.8
≥5,000	39	15.1
Night sleep satisfaction		
Dissatisfaction	70	27.1
Normal	131	50.8
Satisfaction	57	22.1
BMI (kg/m^2^)		
< 18.5	6	2.3
18.5–24	106	41.1
24–28	103	39.9
≥28	43	16.7
KPS score		
≤ 70	44	17.1
80	78	30.2
≥90	136	52.7
Number of courses of chemotherapy		
1–4	123	47.7
5–8	85	32.9
≥9	50	19.4
Tumor stage		
I	52	20.2
II	79	30.6
III	45	17.4
IV	82	31.8
Pathological diagnosis		
Invasive non-specific carcinoma	248	96.1
Other	10	3.9
Average daily step count during the past week		
≤ 4,999	159	61.6
5,000–7,499	87	33.7
≥7,500	12	4.7
Presence or absence of pain		
Absent	122	47.3
Present	136	52.7
Have any other doctor-diagnosed chronic illnesses		
Absent	189	73.3
Present	69	26.7

### 3.2 Classification of latent profile

This study fitted three potential profile models in total. Starting from the initial model 1 and successively increasing the number of model categories, a total of 1 to 4 category models were extracted, and the model fitting results are shown in Table 2. As the number of categories increased, the values of AIC, BIC, and aBIC decreased, and when three categories were retained the Entropy value was 0.930 and the LMRT and BLRT reached the significant level (*P* < 0.05); when four categories were retained, although the Entropy value reached the maximum, the LMRT did not reach the significant level (*P* > 0.05), and the model has a category probabilities below 5%, suggesting that some categories may lack sufficient representation, indicating that the 4-class model is not superior to the 3-class model. Therefore, considering all factors, it is determined that the 3-class model is the best fitting model. The average potential category probabilities for model 3 ranged from 0.952 to 0.991, confirming the high accuracy of the classification.

**Table 2:** Latent profile model fit comparison.

Model	AIC	BIC	aBIC	Entropy	LMRT (*P*)	BLRT (*P*)	Class probability
1	10,015.066	10,121.655	10,026.545				
2	8,930.368	9,093.804	8,947.968	0.897	0.0043	< 0.001	0.512/0.488
3	8,619.717	8,840.000	8,643.440	0.930	< 0.001	< 0.001	0.244/0.392/0.364
4	8,548.356	8,825.487	8,578.201	0.947	0.2169	< 0.001	0.244/0.047/0.372/0.337

**Figure 1:**
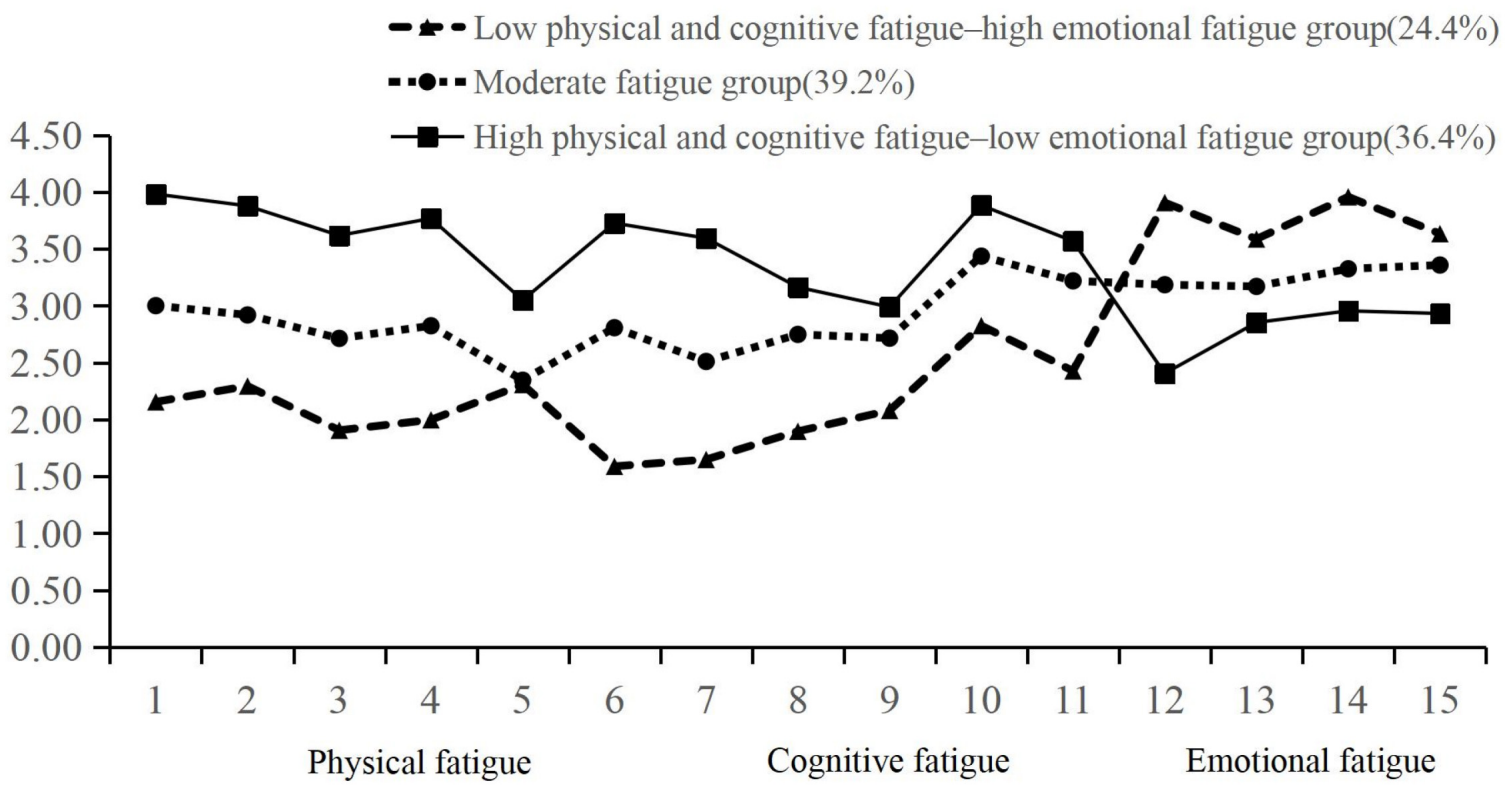
Three latent classes of cancer-related fatigue in patients with breast cancer undergoing chemotherapy. The x-axis represents cancer-related fatigue items. All items of Cancer Fatigue Scale were realigned by dimension, with items 1–7 belonging to physical fatigue dimension, items 8–11 belonging to cognitive fatigue dimension, and items 12–15 belonging to emotional fatigue dimension.

Based on our research findings, we observed three distinct cancer-related fatigue characteristics among patients with breast cancer undergoing chemotherapy. Patients in the Class 1 showed a low probability in the two dimensions of physical fatigue and cognitive fatigue, and reported a higher probability in the dimension of emotional fatigue, so the category was named the low physical and cognitive fatigue–high emotional fatigue group, accounting for 24.4%. Class 2 had moderate response probabilities for nearly all 15 items. Therefore, this category was named moderate fatigue group, which accounted for 39.2%. Class 3 demonstrated a prominently high endorsement probability for all items in the two dimensions of physical fatigue and cognitive fatigue, but low response probabilities in the dimension of emotional fatigue. Therefore, Class 3 was named the high physical and cognitive fatigue–low emotional fatigue group, accounting for 36.4% of the patients. Further details are listed in Figure 1.

### 3.3 Demographic and clinical characteristics of three latent profiles

The differences in demographic and clinical factors among various categories are shown in Table 3. The chi-squared test revealed significant differences in age, working condition, KPS score, number of courses of chemotherapy, tumor stage, average daily step count during the past week, and presence or absence of pain among patients in the different latent profiles (*P* < 0.05).

**Table 3:** Differences in Demographic and clinical characteristics Among the LPA *n* (%).

Item	Low physical and cognitive fatigue–high emotional fatigue group (*n* = 63)	Moderate fatigue group (*n* = 101)	High physical and cognitive fatigue–low emotional fatigue group (*n* = 94)	χ^2^	*P*
Age (years)					
18–44	23 (36.5)	29 (28.7)	16 (17.0)	17.070	0.002
45–59	33 (52.4)	66 (65.3)	57 (60.6)		
≥60	7 (11.1)	6 (5.9)	21 (22.3)		
Education level					
Junior high school and below	37 (58.7)	60 (59.4)	68 (72.3)	4.610	0.330
High school/technical secondary school	16 (25.4)	24 (23.8)	16 (17.1)		
University and above	10 (15.9)	17 (16.8)	10 (10.6)		
Working condition					
Employed	22 (34.9)	30 (29.7)	18 (19.1)	10.718	0.030
Unemployed	34 (54.0)	56 (55.4)	50 (53.2)		
Retired	7 (11.1)	15 (14.9)	26 (27.7)		
Marital status					
Married	56 (88.9)	93 (92.1)	84 (89.4)	0.603	0.740
Other (single/divorced/widowed)	7 (11.1)	8 (7.9)	10 (10.6)		
Family place of residence					
Towns	36 (57.1)	67 (66.3)	55 (58.5)	1.846	0.397
Village	27 (42.9)	34 (33.7)	39 (41.5)		
Per capita monthly household income (¥)					
≤ 2,999	21 (33.3)	31 (30.7)	41 (43.6)	4.991	0.288
3,000–4,999	30 (47.6)	53 (52.5)	43 (45.7)		
≥5,000	12 (19.1)	17 (16.8)	10 (10.7)		
Night sleep satisfaction					
Dissatisfaction	14 (22.2)	26 (25.7)	30 (31.9)	5.845	0.211
Normal	30 (47.6)	51 (50.5)	50 (53.2)		
Satisfaction	19 (30.2)	24 (23.8)	14 (14.9)		
BMI (kg/m^2^)					
< 18.5	3 (4.8)	1 (1.0)	2 (2.1)	3.827	0.700
18.5–24	28 (44.4)	42 (41.6)	36 (38.3)		
24–28	21 (33.3)	41 (40.6)	41 (43.6)		
≥28	11 (17.5)	17 (16.8)	15 (16.0)		
KPS score					
≤ 70	4 (6.4)	8 (7.9)	32 (34.0)	48.827	< 0.001
80	14 (22.2)	27 (26.7)	37 (39.4)		
≥90	45 (71.4)	66 (65.4)	25 (26.6)		
Number of courses of chemotherapy					
1–4	36 (57.1)	53 (52.5)	34 (36.2)	11.463	0.022
5–8	18 (28.6)	34 (33.6)	33 (35.1)		
≥9	9 (14.3)	14 (13.9)	27 (28.7)		
Tumor stage					
I	18 (28.6)	21 (20.8)	13 (13.8)	32.120	< 0.001
II	24 (38.1)	30 (29.7)	25 (26.6)		
III	11 (17.5)	26 (25.7)	8 (8.5)		
IV	10 (15.8)	24 (23.8)	48 (51.1)		
Pathological diagnosis					
Invasive non-specific carcinoma	59 (93.7)	98 (97.0)	91 (96.8)	1.375	0.503
Other	4 (6.3)	3 (3.0)	3 (3.2)		
Average daily step count during the past week					
≤ 4,999	30 (47.6)	54 (53.5)	75 (79.8)	29.599	< 0.001
5,000–7,499	25 (39.7)	44 (43.6)	18 (19.1)		
≥7,500	8 (12.7)	3 (2.9)	1 (1.1)		
Presence or absence of pain					
Absent	36 (57.1)	57 (56.4)	29 (30.9)	16.034	< 0.001
Present	27 (42.9)	44 (43.6)	65 (69.1)		
Have any other doctor-diagnosed chronic illnesses					
Absent	49 (77.8)	76 (75.2)	64 (68.1)	2.145	0.342
Present	14 (22.2)	25 (24.8)	30 (31.9)		

### 3.4 Predictors of three latent profiles

Multivariate logistic analysis was performed using the category of potential profile of cancer-caused fatigue as the dependent variable and items with statistically significant differences in single factor analysis of variance as the independent variables, and the likelihood ratio chi-square value of the final regression model was 97.163 (*P* < 0.001), indicating that the final model was valid, using Class 2 as the reference categories. As shown in Table 4, the results revealed that average daily step count during the past week ≤ 4,999 steps (*OR* = 0.214, *P* = 0.041), KPS score ≤ 70 (*OR* = 6.377, *P* = 0.002) had an increased likelihood of belonging to Class 2. Further, tumor stage III (*OR* = 0.272, *P* = 0.037), absence of pain (*OR* = 0.360, *P* = 0.004) had a likelihood of belonging to Class 3. These findings provide insights into the factors that influence the cancer-related fatigue in patients with breast cancer undergoing chemotherapy, enabling a basis for developing targeted support strategies to reduce the level of fatigue among these patients.

**Table 4:** Multivariate logistic analysis of cancer-related fatigue latent profile.

Variables	Low physical and cognitive fatigue–high emotional fatigue group vs. moderate fatigue group						High physical and cognitive fatigue–low emotional fatigue group vs. moderate fatigue group					
	*B*	*SE*	Wald χ^2^	*P*	*OR*	95% CI	*B*	*SE*	Wald χ^2^	*P*	*OR*	95% CI
Intercept	1.578	1.180	1.787	0.181	–	–	−0.095	1.612	0.003	0.953	–	–
Age (years)										
18–44	−0.676	0.714	0.896	0.344	0.509	0.126–2.061	−1.183	0.683	3.002	0.083	0.306	0.080–1.168
45–59	−1.034	0.650	2.526	0.112	0.356	0.099–1.273	−1.060	0.577	3.372	0.066	0.346	0.112–1.074
Working condition										
Employed	0.428	0.624	0.470	0.493	1.534	0.452–5.207	−0.192	0.587	0.107	0.743	0.825	0.261–2.606
Unemployed	0.208	0.551	0.143	0.706	1.231	0.418–3.629	−0.409	0.464	0.776	0.378	0.665	0.268–1.649
KPS score										
≤ 70	−0.070	0.766	0.008	0.928	0.933	0.208–4.190	1.853	0.604	9.418	0.002	6.377	1.953–20.819
80	0.020	0.443	0.002	0.964	1.020	0.428–2.429	0.775	0.413	3.518	0.061	2.170	0.966–4.877
Number of courses of chemotherapy										
1–4	−0.890	0.760	1.371	0.242	0.411	0.093–1.822	0.264	0.651	0.164	0.686	1.301	0.364–4.659
5–8	−0.830	0.738	1.266	0.260	0.436	0.103–1.851	0.629	0.613	1.054	0.305	1.876	0.564–6.238
Tumor stage										
I	1.176	0.751	2.450	0.117	3.242	0.743–14.141	−0.572	0.681	0.856	0.355	0.564	0.168–1.896
II	0.972	0.701	1.926	0.165	2.644	0.670–10.442	−0.047	0.544	0.007	0.932	0.955	0.329–2.773
III	0.372	0.730	0.260	0.610	1.451	0.347–6.068	−1.301	0.622	4.370	0.037	0.272	0.080–0.922
Average daily step count during the past week										
≤ 4,999	−1.542	0.755	4.171	0.041	0.214	0.049–0.940	1.337	1.360	0.965	0.326	3.806	0.265–54.737
5,000–7,499	−1.471	0.749	3.858	0.050	0.230	0.053–0.997	0.555	1.372	0.164	0.686	1.742	0.118–25.640
Presence or absence of pain										
Absent	−0.034	0.346	0.010	0.922	0.967	0.491–1.905	−1.022	0.352	8.404	0.004	0.360	0.180–0.718

### 3.5 Analysis of different cancer-related fatigue profiles in QoL

In our analysis, we found significant differences in physiological wellbeing, social and familial wellbeing, emotional wellbeing, functional wellbeing, breast cancer-specific concerns and overall QoL among three fatigue profiles (*P* < 0.001). Further pairwise comparisons showed that, except for the score in the Emotional wellbeing dimension, which had no statistically significant difference between Class 1 and Class 2, the differences in the total score of QoL and scores in other dimensions among Class 1, Class 2, and Class 3 were all statistically significant (*P* < 0.001). The results are shown in Table 5.

**Table 5:** Analysis of different cancer-related fatigue profiles in QoL [*M* (*P*25, *P*75)].

Item	Low physical and cognitive fatigue–high emotional fatigue group (C1)	Moderate fatigue group (C2)	High physical and cognitive fatigue–low emotional fatigue group (C3)	Kruskal–Wallis *H* test	*P*	*Post Hoc* Multiple comparisons
Physiological wellbeing	22.00 (20.00, 24.00)	19.00 (17.00, 21.00)	15.00 (13.00, 17.00)	116.728	< 0.001	C1 > C2 > C3
Social and familial wellbeing	17.00 (15.00, 19.00)	13.00 (11.00, 17.00)	12.00 (10.00, 14.25)	43.119	< 0.001	C1 > C2 > C3
Emotional wellbeing	18.00 (16.00, 20.00)	17.00 (15.00, 18.00)	15.00 (12.75, 16.00)	56.167	< 0.001	C1 = C2 > C3
Functional wellbeing	13.00 (11.00, 15.00)	11.00 (8.00, 13.00)	8.00 (6.00, 11.00)	55.730	< 0.001	C1 > C2 > C3
Breast cancer-specific concerns	25.00 (24.00, 27.00)	24.00 (22.00, 26.00)	22.00 (20.00, 25.00)	46.506	< 0.001	C1 > C2 > C3
Overall QoL	93.00 (89.00, 102.00)	84.00 (77.00, 92.00)	72.00 (64.00, 79.25)	111.521	< 0.001	C1 > C2 > C3

## 4 Discussion

### 4.1 LPA of cancer-related fatigue

Although previous studies have identified cancer-related fatigue is a multi-dimensional construct (34, 35), few have used LPA to classify it into clinically significant subgroups among patients with breast cancer undergoing chemotherapy. This study identified three different types of cancer-related fatigue characteristics, named low physical and cognitive fatigue–high emotional fatigue group, moderate fatigue group, and high physical and cognitive fatigue–low emotional fatigue group, indicating significant individual differences in the fatigue characteristics among these patients. Among these profiles, the moderate fatigue group accounted for the highest proportion (39.2%), which is consistent with previous studies (36). In other words, the majority of patients experienced moderate levels of fatigue. Therefore, medical staff should pay attention to the cancer-related fatigue status of patients undergoing chemotherapy and assess their subjective fatigue perception in a timely manner.

The “low physical and cognitive fatigue–high emotional fatigue group” accounted for 24.4%. Patients in this group were more susceptible to emotional problems following chemotherapy, such as depression, loss of interest, sadness, or irritability. They required greater support and care from relatives and friends and sought to relieve emotional stress by sharing their feelings and experiences with others. In contrast, their physical fatigue was relatively mild, with better concentration and clearer thinking. This study suggests that it is urgent to establish supportive care plans based on psychological and social support for these patients. Research has shown that positive psychological and social support can help patients with breast cancer regulate their emotional state, enhance subjective initiative, and thereby minimize cancer-related fatigue (37). Therefore, in addition to providing medical assistance, more spiritual care and psychological comfort should be offered to these patients. Patients should be guided to express their emotions through talking, describing unpleasant events, and other means. Furthermore, family members should be encouraged to spend more time with patients and establish an effective family support system to reduce their level of emotional fatigue.

The “high physical and cognitive fatigue–low emotional fatigue group” accounted for 36.4%. Due to adverse reactions caused by chemotherapy, patients in this group experienced severe physical fatigue, significant decline in physical endurance and activity ability, weakened willingness and ability to participate in social activities, making them more inclined to rest and stay alone. At the same time, cognitive fatigue was prominent, manifesting as decreased memory and slower thinking. Nevertheless, these patients may possess strong psychological resilience and be able to maintain an optimistic attitude to a certain extent. This finding suggests that attention should be paid to this group's complaints regarding the adverse effects of chemotherapy. Assessment and prevention efforts are needed, along with targeted interventions such as lifestyle adjustments and cognitive behavioral therapy, to alleviate their physical and cognitive fatigue.

### 4.2 Demographic and clinical characteristics of patients in different cancer-related fatigue profiles

In this study, patients in different fatigue characteristic groups showed significant differences in demographic and clinical characteristics. Among them, daily steps, KPS score, tumor stage and presence or absence of pain were important factors influencing the characteristics of cancer-related fatigue.

Firstly, patients who walked ≤ 4,999 steps per day, KPS score ≤ 70 during chemotherapy were more likely to be classified into the moderate fatigue group. KPS score ≤ 70 indicates that patients have poor overall physical function and limited ability to perform daily activities (38); while daily step ≤ 4,999 steps directly reflects insufficient daily physical activity and a decreased willingness to engage in physical activity. The reason for this is that these patients may have experienced symptoms such as nausea, vomiting, and muscle aches due to chemotherapy, which led to a reduction in physical activity and a decline in physical function. However, the degree of physical fatigue remains at a relatively moderate level. Additionally, although their emotions and cognition were affected by chemotherapy and the pressure of the disease, they had not yet formed a fatigue pattern dominated by emotional or cognitive fatigue. Therefore, their fatigue state was closer to a moderate fatigue that was relatively balanced in terms of physical, emotional, and cognitive aspects.

Secondly, this study reveals that patients with tumor stage III tend to experience higher levels of physical and cognitive fatigue, while emotional fatigue remains relatively lower. This may be attributed to the fact that these patients often have a significant tumor burden, wherein the rapid proliferation and invasion of tumor cells consume substantial body energy. Concurrently, the accumulation of surgical trauma, chemotherapy side effects (e.g., bone marrow suppression, gastrointestinal reactions, and cardiac-related toxicity), and nutritional deficiencies exacerbates tissue damage and functional decline (39), thereby resulting in pronounced physical fatigue. At the level of disease perception, the complex and demanding treatment process significantly heighten negative emotions (40), which further deplete cognitive resources. Over time, this manifests as decreased attention, impaired memory, and sluggish responses, all of which are symptoms of cognitive fatigue. However, it is worth noting that despite higher levels of physical and cognitive fatigue, these patients exhibit relatively lower emotional fatigue. This seemingly contradictory phenomenon may be due to its strong emotional regulation capabilities. They may be more inclined to adopt positive coping strategies—such as actively seeking social support, focusing on the present moment, and reframing cognitive perspectives—to mitigate the impact of negative emotions. Moreover, a well-established social support system from family, friends, and medical staff may also alleviate emotional stress to some extent, enabling patients to better cope with disease-related challenges and reduce emotional fatigue. These distinct fatigue characteristics suggest that, in clinical practice, more comprehensive and individualized intervention strategies should be developed for for patients with breast cancer with tumor stage III. Such strategies should not only focus on alleviating physical fatigue, protecting and enhancing cognitive function, but also fully utilize and strengthen the social support system to help patients maintain a positive mindset, thereby comprehensively improving their QoL.

Finally, patients who did not report any pain symptoms were more likely to be classified into the high physical and cognitive fatigue group, while simultaneously being assigned to the low emotional fatigue group. Studies have shown that cancer pain is just one of the adverse reactions associated with cancer treatment (41). Even if patients do not exhibit obvious symptoms of pain, the continuous proliferation of tumor and the systemic damage caused by chemotherapy drugs will continue to deplete the body's nutrients and energy, leading to physical function deterioration and subsequently causing physical fatigue. At the same time, the systemic disruptions caused by tumors and chemotherapy continue to affect patients' physical and mental states, interfering with normal cognitive functions and leading to prominent symptoms of cognitive fatigue (42). However, these patients tend to experience lower levels of emotional fatigue, likely because they possess strong psychological adaptation abilities and positive coping patterns that effectively buffer negative emotions and thus maintain a better emotional state.

### 4.3 The QoL of patients in different cancer-related fatigue profiles

The results of this study indicate that there are significant differences in QoL among patients who fall into different potential classifications of cancer-related fatigue. The QoL score showed a gradually declining trend in the three categories of cancer-related fatigue. Patients in the “low physical and cognitive fatigue–high emotional fatigue group” report the highest QoL scores, accompanied by relatively low overall cancer-related fatigue scores. In contrast, patients in the “high physical and cognitive fatigue–low emotional fatigue group” have the lowest QoL scores, but the overall scores of cancer-related fatigue are relatively high. These findings suggest that a lower degree of cancer-related fatigue is associated with a higher QoL, indicating that reducing patients' fatigue levels may exert a positive impact on their QoL, thereby facilitating physical recovery and helping to foster positive psychological qualities.

It is worth noting that this study found that patients with higher levels of emotional fatigue paradoxically reported higher levels of emotional wellbeing. This seemingly contradictory result may be explained by the positive psychological changes experienced by breast cancer patients, combined with the social support they have received. On the one hand, patients who have experienced major traumas such as cancer often re-examine the value of life during post-traumatic growth, achieving positive psychological transformation and personal development (43), thereby gaining a deeper sense of happiness. On the other hand, the social support received by patients itself serves as a powerful positive emotional driver. Consequently, even though the treatment process is accompanied by emotional fatigue, patients can still experience and report higher levels of emotional wellbeing through cognitive restructuring, positive emotion regulation strategies, and the utilization of social resources. This reflects the psychological resilience and adaptability of individuals in the face of adversity.

### 4.4 Implications for nursing practice and research

This study utilized LPA to identify the heterogeneity characteristics of cancer-related fatigue in patients with breast cancer undergoing chemotherapy, providing a basis for implementing precise nursing interventions. Future efforts should focus on developing structured nursing intervention plans tailored to different subgroups based on the classification findings of this study. For patients with “high emotional fatigue,” nursing priorities should shift toward establishing a psychosocial support system to alleviate emotional exhaustion through emotional counseling, mindfulness training, and family support programs. For patients with “high physical and cognitive fatigue,” greater emphasis should be placed on symptom management, including individualized activity plans, cognitive training strategies, and enhanced prevention and symptomatic treatment of chemotherapy-related adverse reactions. Meanwhile, the long-term effects of these intervention plans on improving patients' QoL should be further verified.

### 4.5 Limitation

This study has some limitations. Firstly, this study only conducted a cross-sectional survey on patients with breast cancer undergoing chemotherapy at a tertiary general hospital in this region. The sample may have insufficient representativeness, and it is unable to capture the dynamic trajectories of each variable over time or infer the temporal relationships between variables. Future research should further verify our findings through multi-center surveys and longitudinal studies. Secondly, during the initial design stage of this study, we failed to fully consider the potential impacts of clinical variables such as chemotherapy regimens, chemotherapy cycle timing, targeted therapy, and endocrine therapy on patient cancer-related fatigue and QoL. As a result, we did not specifically collect or control these variables. Future research should collect more comprehensive relevant variables to control residual confounding factors. Finally, in this study, “average daily step count during the past week” was based on patients' self-reports rather than objective measurements such as pedometers or wearable devices. This may lead to recall bias or overestimation or underestimation of the values. Using objective measurement methods such as smart wristbands or pedometers in the future will help improve data accuracy.

### 4.6 Study strength

Based on previous studies, this research has revealed different patterns of cancer-related fatigue in patients with breast cancer undergoing chemotherapy, breaking through the limitations of traditional variable studies and facilitating a cognitive shift from the “group level” to “individual characteristics.” Furthermore, the study not only focuses on different dimensions of chemotherapy-induced fatigue but also links them to outcome such as QoL and emotional wellbeing. The findings have certain guiding value for clinical nursing assessment and intervention.

## 5 Conclusions

In this study, LPA was used to explore latent profiles of cancer-related fatigue in patients with breast cancer undergoing chemotherapy. Three latent profiles were identified: low physical and cognitive fatigue–high emotional fatigue, moderate fatigue, and high physical and cognitive fatigue–low emotional fatigue. The results show that the QoL of patients varies significantly across these different fatigue subtypes. Therefore, it is suggested to implement intervention measures for patients within homogeneous profile of cancer-related fatigue to improve their QoL.

## Data Availability

The datasets presented in this article are not readily available because the data that support the findings of this study are available from the corresponding author [Liping Zhang], upon reasonable request. Requests to access the datasets should be directed to Liping Zhang, 348278186@qq.com.
